# Fracture patterns in cleft orthognathic surgery. a cross-sectional study

**DOI:** 10.21142/2523-2754-1202-2024-194

**Published:** 2024-06-27

**Authors:** Ércio Júnior Montenegro de Andrade, Isabela Toledo Teixeira da Silveira, Bhárbara Marinho Barcellos, Luciano Reis de Araújo Carvalho, Renato Yassutaka Faria Yaedú

**Affiliations:** 1 Hospital for Rehabilitation of Craniofacial Anomalies, University of São Paulo (HRAC/USP). Bauru, Brazil. ercio.montenegro@gmail.com , bharbarambarcellos@usp.br , renatoyaedu@gmail.com Universidade de São Paulo Hospital for Rehabilitation of Craniofacial Anomalies University of São Paulo (HRAC/USP) Bauru Brazil ercio.montenegro@gmail.com bharbarambarcellos@usp.br renatoyaedu@gmail.com; 2 Faculty of Dentistry of Bauru, University of São Paulo, Department of Surgery, Stomatology, Pathology and Radiology. Bauru, Brazil. isabelattsilveira@gmail.com , lucianoreisc@gmail.com Universidade de São Paulo Faculty of Dentistry of Bauru University of São Paulo Department of Surgery, Stomatology, Pathology and Radiology Bauru Brazil isabelattsilveira@gmail.com lucianoreisc@gmail.com

**Keywords:** orthognathic surgery, bilateral sagittal osteotomy of the mandibular ramus, cleft lip and palate, orofacial clefts, cirugía ortognática, osteotomía sagital bilateral de rama mandibular, fisura labiopalatina, fisuras orofaciales

## Abstract

**Objective:**

This study aims to identify fracture patterns on the lingual aspect of the mandible following Bilateral Sagittal Osteotomy of the Mandibular Ramus and correlate these patterns with mandibular anatomical characteristics in patients with cleft lip and palate.

**Methods:**

Two hundred cone beam CT scans were analyzed, with 100 scans in the preoperative period to assess mandibular anatomy and 100 in the postoperative period to evaluate the course of fractures on the lingual surface after surgery.

**Results:**

Statistical analysis revealed no correlation between the depth of the mandibular fossa and the type of fracture after bilateral sagittal osteotomy. Similarly, there was no association between the height and angle of the mandibular body and the type of fracture. The most common fracture type observed was the type 3 pattern, characterized by a line running through the mandibular canal. Furthermore, no relationship was identified between the studied anatomical aspects and the occurrence of undesired fractures.

**Conclusions:**

The anatomical data presented in this study can assist surgeons in selecting the safest surgical techniques and optimal osteotomy sites, particularly in patients with cleft lip and palate.

## RESUMEN

**Objetivo:**

Este estudio tiene como objetivo identificar los patrones de fractura en la superfície lingual de la mandíbula después de la osteotomía sagital bilateral de la rama mandibular y correlacionar estos patrones con las características anatómicas mandibulares en pacientes com fisura labiopalatina.

**Métodos:**

Se analizaron doscientas tomografías computarizadas de haz cónico, con cien tomografías en el período preoperatorio para evaluar la anatomía mandibular y cien en el período postoperatorio para evaluar el curso de las fracturas en la superficie lingual después de la cirugía.

**Resultados:**

El análisis estadístico no reveló correlación entre la profundidad de la fosa mandibular y el tipo de fractura después de la osteotomía sagital bilateral. Del mismo modo, no hubo asociación entre la altura y el ángulo del cuerpo mandibular y el tipo de fractura. El tipo de fractura más común observado fue el patrón tipo 3, caracterizado por una línea que atraviesa el canal mandibular. Además, no se identificó relación entre los aspectos anatómicos estudiados y la ocurrencia de fracturas no deseadas.

**Conclusiones:**

Los datos anatómicos presentados en este estudio pueden ayudar a los cirujanos a seleccionar las técnicas quirúrgicas más seguras y los sitios de osteotomía óptimos, especialmente en pacientes con fisura labiopalatina.

## INTRODUCTION

Despite osteotomy techniques refinements over the years, mandibular separation remains a formidable aspect in orthognathic surgery, with an incidence of *bad splits* ranging from 0.2% to 14.6%^1^. These non-ideal fractures may lead to significant complications, such as nerve injuries, bleeding, challenges in achieving planned movements during orthognathic surgery, and difficulties in stabilizing the mandible, potentially resulting in pseudoarthrosis ^2^. Furthermore, these fractures are associated with bone sequestration, an increased risk of infection, and a higher relapse rate^3^^-^^5^.

In patients with cleft lip and palate (CLP), the craniofacial skeletal configuration often deviates from normal patterns. Consequently, bone fractures induced by mandibular osteotomies may not follow the anticipated course. Notably, the specialized literature lacks studies on fracture patterns and their implications in these patients. Therefore, this study aims to elucidate factors influencing the fracture pattern on the lingual face of the mandible following Bilateral Sagittal Split Osteotomy (BSSO) in patients with CLP. Additionally, the study aims to associate these fracture patterns with the height, angle of the mandibular body, and the depth of the submandibular fossa, with the purpose of determining their influence on the fracture pattern occurring on the lingual face. This knowledge is paramount for the avoidance of bad splits during orthognathic surgery, ensuring enhanced procedural safety, reduced surgical time, minimized blood loss and greater predictability of the final outcome.

## MATERIALS AND METHODS

This study adopted a cross-sectional design. All procedures followed were in accordance with the ethical standards of the responsible committee on human experimentation (institutional and national) and with the Helsinki Declaration of 1975^6^. Informed consent was obtained from all patients for being included in the study and the study was approved by the institution ethics committee under the protocol CAAE: 61600220.0.0000. 

This article evaluated 200 parasagittal images of hemi-mandibles derived from 100 cone beam computed tomography (CBCT) scans in DICOM format. The CBCT scans were sourced from patients with nonsyndromic CLP who underwent orthognathic surgery at the Hospital for Rehabilitation of Craniofacial Anomalies - HRAC/USP. The inclusion criteria encompassed patients of diverse ethnicities and genders, with a mandatory step of BSSO of the mandibular ramus as part of their orthognathic surgery. The surgical procedures could involve mandibular advancement, setback, and/or rotation.

Inclusion Criteria:

• Patients aged 18 years and older.
• Patients who underwent prior orthodontic treatment.
• Patients with nonsyndromic cleft lip and/or palate.
• Patients with Angle Class III occlusion.
• Patients without lesions or artifacts that could alter the morphology of the region.
• Patients with first and second molars in the mandible.
• Patients without third molars in the mandible, and in case of extraction, it should have occurred at least 6 months prior to orthognathic surgery.

The image exams utilized in this study are part of the CBCT scans that were performed during the surgical routine (pre- and post-operative) conducted on patients undergoing orthognathic surgery between the years 2018 and 2022. It is noteworthy that these scans were already part of the standard procedure for these patients, and no additional tests were conducted solely for the purpose of this study. Patients included in the sample were contacted via telephone, and an email with an online informed consent form was signed for authorization.

### TOMOGRAPHIC EVALUATION

The day after surgery, as part of the routine, study participants underwent a computed tomography cone beam exam.

For the *mandibular anatomy evaluation*, the CBCT scans were imported into Dolphin Imaging 11.8 Premium, and the "Build X-Rays" tool was selected. Subsequently, the "Cross Section Lower" tool was utilized to demarcate only the mandibular region and position the lines of the cross-section between the molars. The mandibles were oriented so that the occlusal plane remained parallel to the ground and perpendicular to the reference line provided by the program.

**Figure 1:**
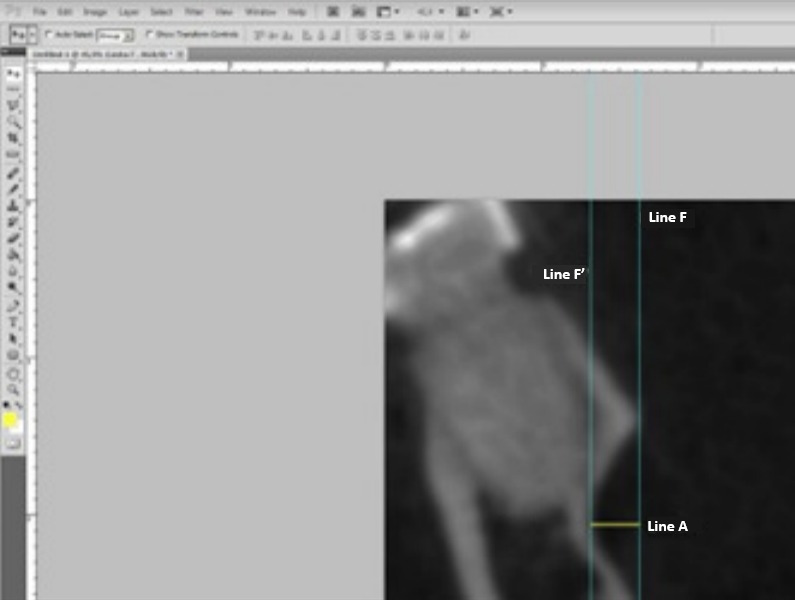
Measurement of the depth of the mandibular fossa. Lines F and F' tangentially touch the innermost and outermost points of the fossa, and Line A refers to the depth.

Measurements were taken by establishing specific reference points, such as the upper and lower most prominent points of the lingual concavity corresponding to the delineation of the mandibular fossa. The A line was drawn between these points to define the end of the mandibular fossa. After drawing this line, the "Straighten" tool in Adobe Photoshop CS6 was used to make it perpendicular to the ground. However, this line did not remain visible, making necessary the drawing of two additional lines perpendicular to the ground, marking the beginning (line F) and the end (line F ´) of the fossa. The measurement of the mandibular fossa (line A) was the result of the distance between lines F and F ´, ensuring they were perpendicular to each other for greater measurement accuracy (Figure 1).

### MORPHOLOGICAL CLASSIFICATION

**Figure 2:**
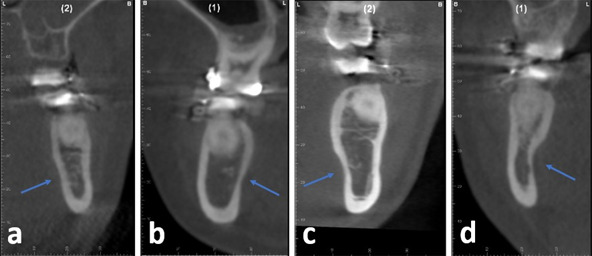
Morphological classification based on the measurement of the depth of the mandibular fossa. (a) Type a; (b) Type b; (c) Type c; and (d) Type d.

The morphological classification was based on the measurement of the mandibular fossa depth, as described above, and categorized into four types (Figure 2):

• Type a: Distance between 0 and 1 mm
• Type b: Distance between 1.1 and 2 mm
• Type c: Distance between 2.1 and 3 mm
• Type d: Distance greater than 3.1 mm

#### Mandibular Heights Measurement

**Figure 3:**
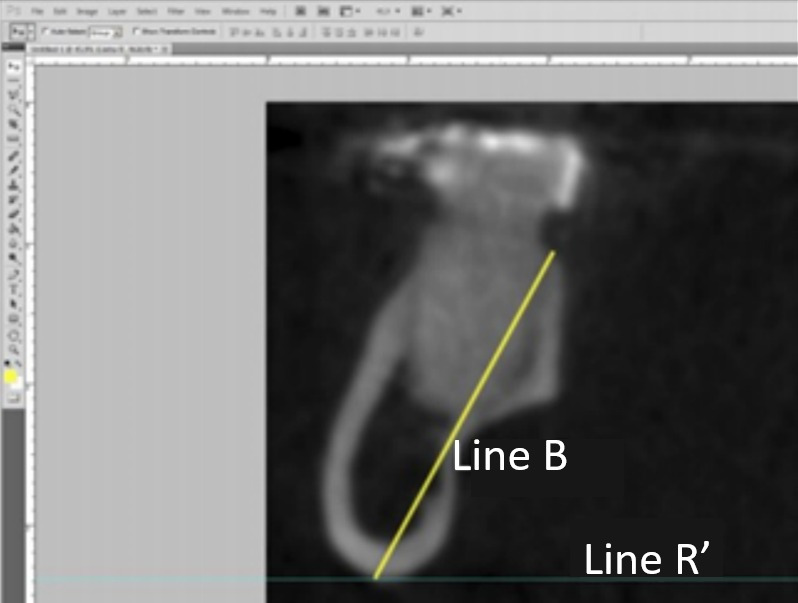
Measurement of mandibular height. Line R' tangentially touches the lowest point of the mandibular base, and Line B refers to the height.

The heights of the mandibles were also measured using a line that starts from the lingual portion of the alveolar ridge to the lowest point of the mandibular base (Line B) (Figure 3).

**Figure 4:**
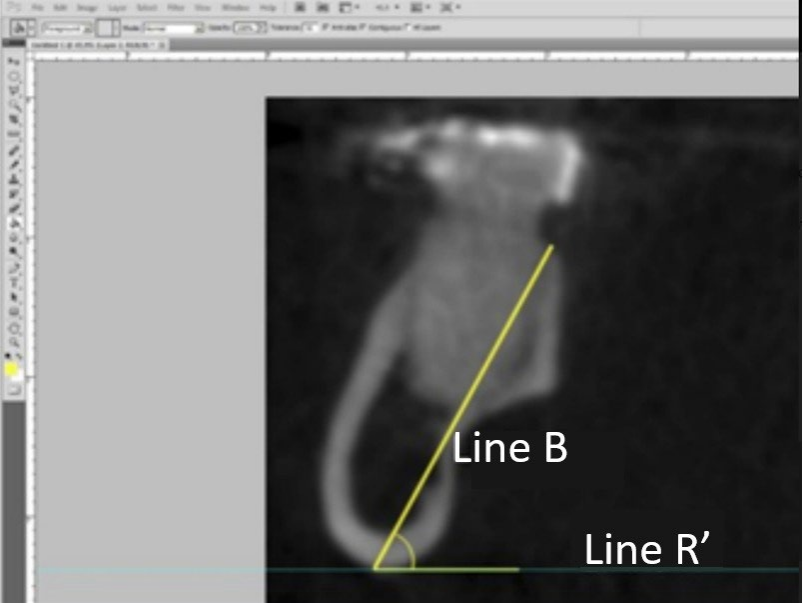
Measurement of the mandibular angle between Line R' and Line B.

B Line was then used to measure the angle of the mandibular body, with the angle formed between it and a second line parallel to the ground (Line R') (Figure 4).

Mandibular anatomy was classified based on the visualization of the transverse section of the mandibular ramus between the first and second molars, using the methodology of Parnia et al. (2010) ^(^^7^. This classification categorizes mandibles into three types:

• Type I: Lingual concavity less than 2 mm;
• Type II: Lingual concavity between 2 and 3 mm;
• Type III: Lingual concavity greater than 3 mm.

For the tomographic evaluation of the induced fracture on the lingual surface after orthognathic surgery, the mandible was digitally isolated from the maxilla and the cranial base. It was then divided along the midline in the CBCT. The left and right sides of the mandible were rotated along the vertical axis to visualize the vestibular and lingual surfaces of the mandibular ramus three-dimensionally in axial, sagittal, and coronal planes, in both lateral and medial views. The step aimed to categorize different fracture patterns.

The scale used in this study was developed by Plooij (2009)^8^, which consists in four categories based on the trajectory of the fracture line on the lingual face of the mandibular ramus. The classification of the fracture was determined by the trajectory it follows on the lingual face, starting from the extension of the mid-ramus osteotomy, following one of the fracture paths:

• Type 1: The fracture occurs through or behind the mandibular foramen and extends toward the inferior border of the mandible, as described by Hunsuck (1968) ^8^.
• Type 2: The fracture follows the bone medially and extends toward the posterior border before bending towards the inferior border of the ramus.
• Type 3: The fracture follows through the mandibular foramen and the mandibular canal towards the inferior border.
• Type 4: This type encompasses all other patterns of undesired fractures or bad splits.

To assess the influence of the final position of the medial bone cut on the path of the lingual fracture line, the medial bone cuts were classified using the anterior edge of the mandibular foramen as the starting point.

*Statistic analysis*

All data were tabulated in a Google Spreadsheet and organized for statistical analysis using the Sigma Plot 12.0 program. 

Intra-examiner calibration was performed using 30% of the sample from each group with repetition after a 15-day interval. The ICC was calculated in Google Spreadsheet.

Statistical tests included the Shapiro-Wilk test, Chi-square test, and Pearson correlation. These tests were used to analyze the distributions of fracture types based on the anatomical classification of the mandible in the preoperative period.

## RESULTS

For this study, a total of 200 facial CBCT scans were evaluated, with 100 scans taken preoperatively and 100 postoperatively, obtained from patients operated on between 2018 and 2022.

**Figure 5:**
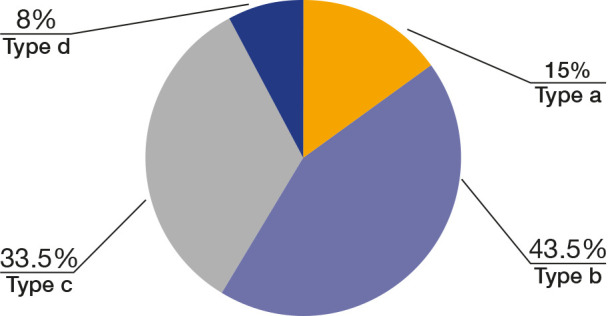
Distribution of participants based on the depth pattern of the mandibular fossa.

**Figure 6:**
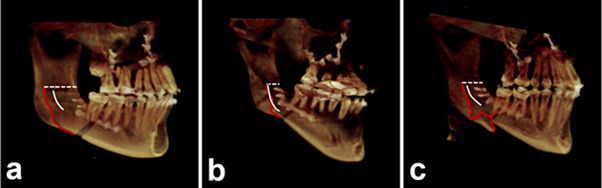
The types of fracture patterns found in this study, according to Plooij (2009) classification; (a) Type 1, (b) Type 3 e (c) Type 4.

**Figure 7:**
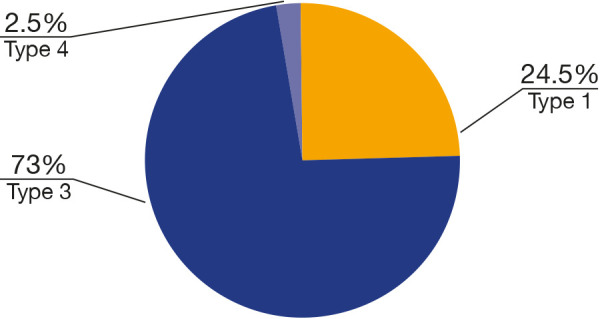
Distribution of participants according to the pattern of depth of the mandibular fossa.

The selected patients for the study encompassed 43 men (43%) and 57 women (57%), with an average age ranging from 23 to 35 years. In this study, 87 mandibles (43.5%) exhibited the mandibular fossa depth categorized as Type b, which was the predominant pattern, followed by types c (33.5%), a (15%), and d (8%) (Figure 5). No significant relationship was found between mandibular fossa depth and mandibular body height (p = 0.845). There was a statistically significant difference between mandibular fossa depth and the mandibular body angle (p = 0.006). Concerning the types of fractures proposed by Plooij (2009) ^7^, 146 patients (73%) presented type 3 fractures, followed by 49 (24.5%) patients with type 1 and 5 (2.5%) with type 4 (Figure 6). No type 2 fractures were observed (Figure 7).

Furthermore, there is no relationship between the angle and different types of fractures (p = 0.691). Also, there is no relationship between the fracture type and mandibular fossa depth (p = 0.643). There is no relationship between mandibular body height and the resulting fracture type (p = 0.117) and there is no relationship between the side of the mandible and the type of fracture after osteotomy (p = 0.920). The Pearson correlation test showed that the variables mandibular fossa depth and mandibular body height had a positive correlation. This implies that the greater the mandibular fossa depth, the greater the mandibular body height. However, the variables mandibular body angle and mandibular fossa depth had a negative correlation, suggesting that a greater angle corresponds to a smaller mandibular fossa depth. The variables mandibular body angle and mandibular body height did not show a correlation with each other.

## DISCUSSION

Orthognathic surgery in patients with CLP presents unique challenges that require specific measures regarding the surgical techniques used in these patients. However, the literature lacks studies that explore mandibular anatomy related to anatomical features of the mandible, even in patients without CLP. This study continues the investigation and evaluation of the possible influence of mandibular body aspects, the region where the bilateral sagittal osteotomy of the mandibular ramus is performed, on the occurrence of different types of fracture, with the intention to avoid *bad splits*.

Hou et al. (2015) ^(^^9^ evaluated cortical bone thickness at the posterior border of the mandibular ramus, the degree of mandibular angulation, and the shape of the mandibular ramus through axial planes of CBCT scans. They suggested that these factors influence the resulting fracture pattern after osteotomy in orthognathic surgery. However, they did not find a significant association between the fracture pattern and the age and gender of the patients, which is consistent with this study that also did not find an association between gender, age, mandibular morphological patterns and fracture types.

The study by Mello (2017) ^10^, which found no differences between angulation and mandibular body height and mandibular fossa depth when comparing CT scans of patients with and without CLP. Either way, the mandibular fossa is an anatomical feature of great importance when evaluating the positioning of the mandibular canal, in addition to the thickness and angulation of the mandibular body. Furthermore, studies focus on possible anatomical factors of the mandible that may cause undesired fractures, but they do not provide a correlation between possible fracture patterns and the morphological peculiarities of the operated mandibles ^11^^-^^15^. Thus, there is no scientifically grounded surgical predictability.

The present study suggests that the type of fracture may not be determined by the specific mandibular anatomical characteristics studied. Despite no relationship between the side of the mandibular body and the fracture, and mandibular body height not affecting the resulting fracture type, it is essential to consider that hemi-mandibles are not symmetrical. Differences in mandibular body heights and angles and fossa depth may require different surgical planning regarding the direction of the osteotomy cut.

The direction of the osteotomy in the mandibular body can be influenced by the anatomy of the region, considering that the height and angle of the mandibular body and fossa depth may affect the deviation of the cut to avoid reaching the inferior alveolar nerve. Moreover, deviation from the cut may lead to a *bad split* and compromise the thickness of one of the bone plates which complicates its correct positioning during fixation ^16^^,^^17^. According to the study by Tomomatsu and colleagues (2021) ^(^^18^, a sharp angle of the mandibular ramus can result in a shallower cut during osteotomy, increasing the likelihood of a *bad split*. Thus, to perform osteotomy in a mandible with a sharper angle, it may be necessary to deepen the saw and/or drill at the time of the cut to ensure that it reaches properly the base of the mandible.

There is a significant variability in the morphology of the cross-sectional area between patients with and without CLP related to the angle of the mandibular body ^10^. It is believed that the CLP can influence the development of the mandible concerning its angulation. This study demonstrated a possible negative correlation between the angle of the mandibular body and mandibular fossa depth. Furthermore, it was observed that the more open the angle, the higher the chance of pattern A mandible fossa since a larger angle corresponds to a shallower fossa. However, even though the fracture type does not correlate with the angle, fracture patterns may occur due to other factors not considered in this study, such as the thickness of the mandibular body.

Wang (2016) ^19^ suggests that patients with low thickness in the vestibulo-lingual region distal to the second molar (body region) had a higher risk of undesired fractures, which fall into pattern type 4 ^7^. In a study conducted on patients without CLP ^11^, the authors obtained a *bad split* rate of 14.6%. When relating these data to the measurement of mandibular thickness and the height of the mandibular body in the molar region, they found that when the vestibulo-lingual thickness of the mandible was less than 10.17 mm, 98.8% of patients in this group presented a *bad split*.

Although mandibular thickness was not measured in this study, it can be considered that the deeper the mandibular fossa in the region, the thinner the mandibular body. This assertion is supported by the study of Cunha et al. (2020) ^12^, where the authors measured mandibular thickness and related it to the fracture pattern, as established by Plooij (2019) ^(^^7^. After the evaluations, they found that 56.5% of fractures were type 1, and 11.3% were type 4. Most type 4 fractures were associated with lower bone thickness, while in type 1, where the division occurs just behind the mandibular canal, the mandibles had a considerable amount of medullary bone, indicating greater bone thickness^(7, 12)^.

The measurements regarding the depth of the mandibular fossa in this sample exhibited a normal distribution, even though two mandibles had a measurement of 4.6 mm, the highest value recorded. Despite this extreme, these patients experienced a type 3 fracture, which is not considered a *bad split*, demonstrating that the depth of the mandibular fossa does not directly correlate with the occurrence of *bad splits*, as observed in the statistical analysis.

Furthermore, it is essential to emphasize that changes in the surgical technique can alter the fracture behavior. In Plooij's study (2009) ^(^^7^, 51.3% of fractures were type 1, while type 3 represented 32.5% of the sample. In our study, type 3 (the fracture extends through the mandibular canal) was the most prevalent (73%). This difference may be related to the surgeon and the surgical technique used, considering that qualitatively, fractures of types 1 and 3 do not overlap ^7^.

In this sample, type 2 fractures were not observed, with type 3 being the most present. In contrast, in the study by Hou, Yu, and Wang (2015), no fractures were observed through the mandibular canal (type 3), with fractures divided into two types: the majority (75.38%) occurred on the lingual side of the mandibular ramus near the mylohyoid groove, while the remaining 24.62% occurred at the posterior edge of the mandibular ramus, consistent with type 2 of Plooij (2009) ^7^. This finding reinforces that the type of fracture may also be related to the surgeon's technique and level of experience ^9^.

Thinner cortical bone is biomechanically less resistant in the osteotomy region, making it easier to split during cleavage with chisels ^20^. Kim and Park (2012) ^(^^21^ reported that lingual cortical bone is thinner than buccal cortical bone. Thus, osteotomy fractures are more commonly observed on the lingual side of the mandible, mostly in the region of the mandibular canal due to thinner thickness and greater fragility. The higher occurrence of type 3 fractures may be related to the mandibular canal being an area of lower resistance. Therefore, an essential point for discussion is the fulcrum created during the separation of the mandibular segment, which may be adjusted when areas of greater resistance are encountered ^22^, preventing undesired fractures. In general, fractures usually occur in a region weaker in terms of structure and biomechanics ^21^^,^^22^.

A type 3 fracture may increase the risk of inferior alveolar nerve injury with postoperative sensory repercussions, as the fracture line passes through the mandibular canal, increasing the risk of nerve exposure and manipulation during surgery. Verweij (2015) ^23^, through the evaluation of imaging exams of 176 patients, found an average mandibular body height of 22.7 mm and demonstrated that low mandibular body height (< 22 mm) can significantly increase the risk of inferior alveolar nerve hypoesthesia. In this study, the average mandibular body height was 28.46 mm, a considerably higher value than Verweij's study (2015) ^23^, which may have influenced the low rate of undesired fractures. Additionally, Yoshioka et al. (2010) ^24^ found that a thinner vestibular wall of the mandibular body increases the chances of inferior alveolar nerve sensory disturbances after orthognathic surgery in patients without clefts.

Preoperative CBCT images allow for specific planning for each patient by evaluating the morphological anatomical patterns of the mandible, paying attention to the angulation of osteotomies, and predicting probable fractures that may occur during surgery. While most studies primarily address osteotomies related to mandibular anatomical patterns, focusing on possible characteristics that may lead to bad splits ^11^^,^^18^^,^^19^^,^^25^, they do not necessarily explore different fracture patterns that may occur and do not fall into the category of bad splits. Similar to Plooij's study (2009) ^7^, the number of undesired fractures also represented 2.5% of the total sample in this work. In this sample, type 4 fractures were related to morphological classifications B (60%) and C (40%) of mandibular fossa depth.

The literature emphasizes the importance of mandibular fossa depth for dental implants, stating that a more pronounced concavity may increase the risk of lingual cortical perforation during implant placement6. Chicrala (2014) ^26^ studied the influence of mandibular fossa depth on mandibular anatomy in patients without CLP, showing that a deeper fossa results in greater morphological changes in the lingual contour of the mandible, which may increase the risks of complications in dental surgeries. However, when related to orthognathic surgery in patients with CLP, this anatomical aspect is often overlooked in assessing the success of osteotomies and predicting the resulting fracture pattern.

The literature reports that the presence of impacted third molars, patient age, surgical technique employed, duration of osteotomy, a narrower distance from the mandibular canal to the vestibular cortex, incomplete osteotomy of the inferior border, variations in mandibular anatomy, and lack of surgeon experience may represent risk factors for *bad splits*^11^^,^^12^^,^^15^^,^^23^^,^^27^^-^^32^. However, none of these factors have strong scientific evidence supporting their role in *bad splits* occurrence.

### Limitations

This study aimed to address possible occurrences in orthognathic surgeries in patients without clefts that can be compared and applied to patients with CLP. Although the comparison of studies and samples was possible, it is essential to consider with caution that patients with CLP require an individualized and specific approach due to the craniofacial morphological characteristics they present. Therefore, specialized groups in the rehabilitation of patients with cleft lip and palate should conduct further research on anatomical patterns related to the behavior of these patients when undergoing orthognathic surgery.

## CONCLUSION

There is no relationship between the depth of the mandibular fossa and the type of fracture after BSSO; there is no relationship between the height of the mandibular body and the type of fracture after BSSO; there is no relationship between the angle of the mandibular body and the type of fracture after BSSO; the most common fracture type is pattern type 3, where the fracture line runs through the mandibular canal; there is no relationship between the anatomical aspects studied and the occurrence of undesired fractures; Finally, the anatomical data presented can assist surgeons in choosing the safest surgical techniques and optimal osteotomy sites in patients with CLP.
